# Effect of Systemic Lupus Erythematosus on the Risk of Incident Respiratory Failure: A National Cohort Study

**DOI:** 10.1371/journal.pone.0163382

**Published:** 2016-09-21

**Authors:** Jun-Jun Yeh, Yu-Chiao Wang, Jiunn-Horng Chen, Wu-Huei Hsu

**Affiliations:** 1 Ditmanson Medical Foundation Chia-Yi Christian Hospital, Chiayi, Taiwan; 2 Chia Nan University of Pharmacy and Science, Tainan, Taiwan; 3 Meiho University, Pingtung, Taiwan; 4 Management Office for Health Data, China Medical University Hospital, Taichung, Taiwan; 5 College of Medicine, China Medical University, Taichung, Taiwan; 6 Graduate Institute of Clinical Medicine Science, College of Medicine, China Medical University, Taichung, Taiwan; 7 Division of Rheumatology, Department of Internal Medicine, China Medical University Hospital, Taichung, Taiwan; 8 Division of Pulmonary and Critical Care Medicine, Department of Internal Medicine, China Medical University Hospital, Taichung, Taiwan; Peking University First Hospital, CHINA

## Abstract

**Purpose:**

We conducted a nationwide cohort study to investigate the relationship between systemic lupus erythematosus (SLE) and the risk of incident respiratory failure.

**Methods:**

From the National Health Insurance Research Database, we identified 11 533 patients newly diagnosed with SLE and 46 132 controls without SLE who were randomly selected through frequency-matching according to age, sex, and index year. Both cohorts were followed until the end of 2011 to measure the incidence of incident respiratory failure, which was compared between the 2 cohorts through a Cox proportional hazards regression analysis.

**Results:**

The adjusted hazard ratio (aHR) of incident respiratory failure was 5.80 (95% confidence interval [CI] = 5.15–6.52) for the SLE cohort after we adjusted for sex, age, and comorbidities. Both men (aHR = 3.44, 95% CI = 2.67–4.43) and women (aHR = 6.79, 95% CI = 5.93–7.77) had a significantly higher rate of incident respiratory failure in the SLE cohort than in the non-SLE cohort. Both men and women aged <35 years (aHR = 31.2, 95% CI = 21.6–45.2), 35–65 years; (aHR = 6.19, 95% CI = 5.09–7.54) and ≥65 years (aHR = 2.35, 95% CI = 1.92–2.87) had a higher risk of incident respiratory failure in the SLE cohort. Moreover, the risk of incident respiratory failure was higher in the SLE cohort than the non-SLE cohort, for subjects with (aHR = 2.65, 95% CI = 2.22–3.15) or without (aHR = 9.08, 95% CI = 7.72–10.7) pre-existing comorbidities. In the SLE cohort, subjects with >24 outpatient visits and hospitalizations per year had a higher incident respiratory failure risk (aHR = 21.7, 95% CI = 18.0–26.1) compared with the non-SLE cohort.

**Conclusion:**

Patients with SLE are associated with an increased risk of incident respiratory failure, regardless of their age, sex, and pre-existing comorbidities; especially medical services with higher frequency.

## Introduction

Respiratory failure is a common cause of admission to intensive care units [1]. Respiratory failure can be divided into 1) acute respiratory failure (ICD-9-CM code 518.81) which is hypoxaemic (arterial oxygen tension, PaO_2_ < 60 mmHg) with or without hypercapnia (arterial carbon dioxide tension, PaCO_2_ > 50 mmHg), develops within minutes or hours (pH <7.3 and the value of the bicarbonate ion is normal) in patients with loss of the ability to ventilate adequately or to provide sufficient oxygen to the blood and systemic organs; 2) chronic respiratory failure (ICD-9-CM code 518.83) which is hypercapnic and hypoxaemic, develops over several days or longer (normal or slightly decreased pH and the value of the bicarbonate ion increased) in patients with existing respiratory disease; and 3) acute on chronic respiratory failure (ICD-9-CM code 518.84) which is an acute deterioration in an individual with chronic respiratory failure (PaCO_2_ > 50 mmHg, pH <7.3 and the value of the bicarbonate ion increased; hypoxaemic) [2–4].

Systemic lupus erythematosus (SLE) is more prevalent in women, particularly of child-bearing age [5]. A recent population-based study in Taiwan reported that the average incidence of SLE cases between 2003 and 2008 was 4.87 per 100 000 person-years [5]. SLE is an autoimmune disease that affects multiple organ systems [6] such as the heart, joints, skin, lungs [7,8], chest wall, pleura, vessels [9], liver, kidneys [10], and nervous system [11]. SLE is associated with an increased risk of incident lung diseases (e.g., obstructive airway disease [7,12], pneumonia [8], and pulmonary embolism [13]), pleurisy and pleural effusion [14], and incident atherosclerosis-related diseases (e.g., hypertension [15], diabetes [16], stroke [15,17], and end-stage renal diseases; ESRD [18]). These SLE complications may lead to acute hypoxaemic failure (e.g., pneumonia [19]) with or without hypercapnia (e.g., asthma [7]), chronic respiratory failure (e.g., chronic obstructive pulmonary disease; COPD [12]), or acute on chronic respiratory failure (e.g., interstitial lung disease [14], stroke with hyperlipidemia [20], and ESRD [21] with alveolar edema) [3].

**Fig 1:**
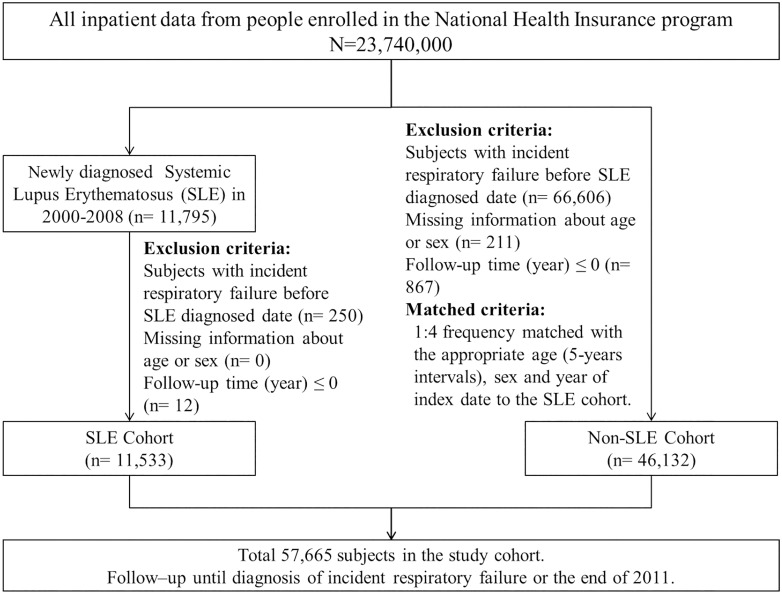
Flowchart presenting the process of selecting the study subjects.

Young patients with the onset of SLE, stroke, hypertension, or ESRD have a poor prognosis [22]. Patients with SLE may exhibit severe deterioration of respiratory function such as incident respiratory failure [23]. Deterioration of major organ function may be directly caused by the disease [24], even in patients without pre-existing comorbidities [25]. We hypothesized that SLE is associated with an increased risk of lung [26] or cardiovascular diseases [20] or exacerbates pre-existing airway or vascular diseases [7,12,13], thus contributing to incident respiratory failure (Fig 1). To the best of our knowledge, this is the first English-language study evaluating the relationship between SLE and the risk of incident respiratory failure in the general population.

## Material and Methods

### Data source

Taiwan’s National Health Insurance (NHI) program is a universal insurance program that was launched in 1995. Enrollment is mandatory to ensure adequate risk pooling and broad-based fund collection. Since 2007, the NHI has covered approximately 99% of Taiwan’s population [27]. The National Health Research Institutes (NHRI) obtained the enrollment files and original reimbursement claims data of NHI enrollees to build the National Health Insurance Research Database (NHIRD). The NHRI releases information for research in medicine and health care. We used 2 types of data from the NHIRD in the current study: the Registry for Catastrophic Illness Patient Database (RCIPD) and the inpatient claims data of NHI enrollees from 1997 to 2011. The NHRI protects personal information in the NHIRD by encrypting the identification codes of patients and medical facilities before releasing the data. The diagnoses of diseases in the NHIRD are based on International Classification of Diseases, Ninth Revision, Clinical Modification (ICD-9-CM) codes.

### Study population

Fig 1 presents a flow chart of the selection process. This study used a population-based cohort study design [28] and ICD-9-CM codes to define the status of SLE (ICD-9-CM code 710.0) [29], and incident respiratory failure (ICD-9-CM codes 518.81, 518.83, and 518.84). In Taiwan, patients with chronic inflammatory diseases, including those with SLE, can apply for a catastrophic illness certificate; in addition, patients with SLE who fulfill 4 or more diagnostic criteria based on the American College of Rheumatology criteria are eligible for this certificate [13]. The 11 criteria are malar rash; discoid rash; photosensitivity; oral ulcers; nonerosive arthritis; pleuritis or pericarditis; renal disorder, persistent proteinuria, or cellular casts in the urine; neurologic disorder, seizures, or psychosis; hematologic disorder, including hemolytic anemia, leukopenia, lymphopenia, or thrombocytopenia; the production of antinuclear antibody (Ab); and other immunologic disorders, including the production of anti-DNA, anti-Smith or antiphospholipid Abs. Patients with an application showing a catastrophic illness certificate are exempted from copayments. All certificate applications are scrutinized through peer review. We used the RCIPD to identify 11 533 patients with incident SLE in the claims data between 2000 and 2008; the date of SLE diagnosis was used as the index date [30]. For each patient with SLE, 4 controls frequency-matched by age (5-y intervals), sex, and index year were selected from patients without SLE (n = 46 132) to comprise the non-SLE cohort. The dates of randomly selected outpatient or inpatient visits during the index years were selected as the index dates for the non-SLE cohort. Patients were excluded if they had experienced incident respiratory failure before the index date (SLE diagnosis date) or had missing data. We analyzed the development of incident respiratory failure in the SLE cohort compared with that in the non-SLE cohort. The follow-up person-years were calculated at the end of 2011 for each patient or until the diagnosis of incident respiratory failure (n = 1182), withdrawal from the insurance system (n = 3256; 5.65%), or death (n = 18 683; 3.24%). Adjustment was made for pre-existing comorbidities, namely hypertension (ICD-9-CM codes 401–405), hyperlipidemia (ICD-9-CM code 272), diabetes (ICD-9-CM code 250) [31], COPD (ICD-9-CM codes 491, 492, and 496), asthma (ICD-9-CM codes 493 and 494), stroke (ICD-9-CM codes 430–438), ischemic heart disease (IHD; ICD-9-CM codes 410–414) [32], pneumonia (ICD-9-CM codes 480–487) [33,34], ESRD (ICD-9-CM code 585), and pulmonary embolism (ICD-9-CM code 415.1), present before the index date.

We used propensity score matching [35] to confirm the results. In addition, we corrected for various risk factors, including sex, age, hypertension, hyperlipidemia, diabetes, COPD, asthma, pneumonia, stroke, ESRD, IHD, pulmonary embolism, and index year, in both cohorts at a 1:3 ratio.

### Ethics Statement

The NHIRD encrypts patients’ personal information to protect their privacy and thus provides researchers with anonymous identification numbers associated with relevant claims information, including sex, birth date, medical services received, and prescriptions. Therefore, patient consent is not required in order to access the NHIRD. This study was approved to fulfill the condition for exemption by the Institutional Review Board (IRB) of China Medical University (CMUH104-REC2-115). The IRB also specifically waived the consent requirement.

### Data Availability Statement

All data and related metadata were deposited in an appropriate public repository (http://nhird.nhri.org.tw/en/index.html). The study population data obtained from the NHIRD (http://nhird.nhri.org.tw/en/index.html) are maintained by the NHRI (http://nhird.nhri.org.tw/), a nonprofit foundation established by the Taiwan government. Only citizens of Taiwan who fulfill the requirements of conducting research projects are eligible to apply for data from the NHIRD. The use of the NHIRD is limited to research purposes only. Applicants must follow Taiwan’s Computer-Processed Personal Data Protection Law (http://www.winklerpartners.com/?p=987) and related regulations of the National Health Insurance Administration and NHRI, and an agreement must be signed by the applicant and his or her supervisor upon application submission. All applications are reviewed for approval prior to data release.

### Statistical Analysis

We used the chi-squared test to compare demographic characteristics such as sex, age (< 35, 35–65, and ≥ 65 y), and history of comorbidities between the SLE and non-SLE cohorts. The mean age of the patients in both cohorts was analyzed using the Student *t* test. A Poisson regression model was used to estimate the incidence rate ratio (IRR) and 95% confidence interval (CI) of incident respiratory failure in both cohorts. The crude hazard ratios (HRs) of patients with SLE and those without SLE were measured using Cox proportional hazards regression models. After adjustment for potential risk factors (i.e., age, sex, hypertension, hyperlipidemia, diabetes, COPD, asthma, pneumonia, stroke, IHD, pulmonary embolism, and ESRD), the risk was presented as an adjusted HR (aHR) of the SLE cohort by using multivariable Cox proportional hazards regression and compared with that of the non-SLE cohort. The continuous variable of age and dichotomous variables of sex, hypertension, hyperlipidemia, diabetes, COPD, asthma, pneumonia, stroke, IHD, pulmonary embolism, and ESRD were included in the multivariable Cox proportional hazards regression. In addition, the association between the number of medical visits (outpatient visits and hospitalizations) per year resulting from SLE exacerbation and the risk of respiratory failure were calculated. The cumulative respiratory failure incidence rate was calculated for each cohort. Kaplan–Meier analysis was used to estimate the cumulative incidence rate of incident respiratory failure for each subgroup, and a log-rank test was performed to evaluate any between-groups differences.

Propensity scores [35] were calculated using logistic regression to estimate the probability of SLE assignment according to the baseline variables including year of SLE diagnosis, sex, age, and history of comorbidities. After propensity score matching, we performed Cox proportional hazards model stratification of matched pairs to estimate differences in the risk of incident respiratory failure between the SLE and non-SLE cohorts. All statistical analyses were performed using the Statistical Analysis Software Version 9.4 (SAS Institute Inc., NC, USA); a 2-tailed *P* value of < .05 was considered statistically significant.

## Results

**Fig 2:**
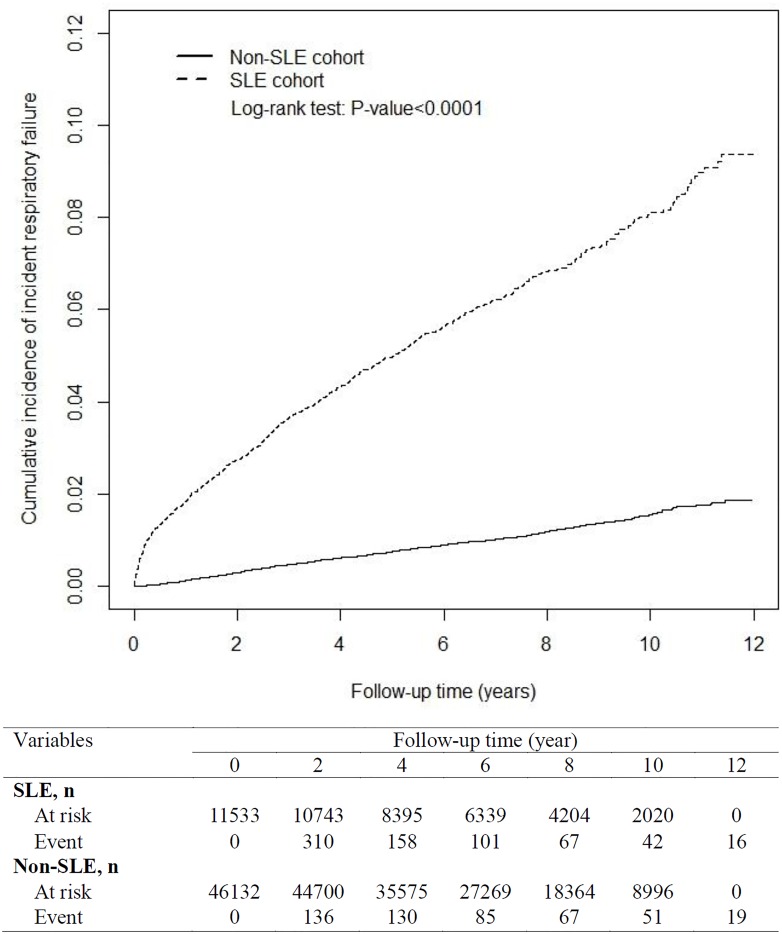
Cumulate incidence of incident respiratory failure between the SLE and non-SLE cohorts, obtained using the Kaplan–Meier model.

Table 1 shows the demographic characteristic variables in the 2 cohorts. Both cohorts had similar sex and age distributions (chi-squared test: *P* = .99) and higher proportions of women (88.0%) and patients older than 35 years (53.2%). The mean age of the patients was 35.9 years in both cohorts (Student *t* test: *P* = .93)[36]. The SLE cohort also included patients with childhood onset of SLE, and the number of SLE patients younger than 10 years was 139. Compared with the non-SLE cohort, the SLE cohort had a higher percentage of patients with hypertension (2.61% vs. 7.98%), hyperlipidemia (0.74% vs. 2.28%), diabetes (1.64% vs. 2.19%), COPD (0.41% vs. 0.79%), asthma (0.72% vs. 1.23%), stroke (1.09% vs. 2.46%), IHD (2.35% vs. 4.13%), pneumonia (1.77% vs. 7.46%), ESRD (0.17% vs. 0.64%), and pulmonary embolism (0.09% vs. 1.07%; chi-squared test: *P* < .0001). The mean follow-up period was 6.84 years for the non-SLE cohort and 6.47 years for the SLE cohort. By the end of the follow-up period, the cumulative incidence of incident respiratory failure was higher in the SLE cohort than in the non-SLE cohort (Fig 2).

**Table 1:** Comparison of the demographics and health status between the SLE and non-SLE cohorts at baseline.

	SLE				
	No (N = 46132)		Yes (N = 11533)		*p value*
Variables	n	%	n	%	
**Sex**					0.99
Women	40592	88.0	10148	88.0	
Men	5540	12.0	1385	12.0	
**Age, year**					0.99
<35	24532	53.2	6133	53.2	
35–65	18608	40.3	4652	40.3	
≥65	2992	6.49	748	6.49	
Mean (SD) ^#^	35.9 (16.3)		35.9 (16.2)		0.93
**Comorbidity**					
Hypertension	1202	2.61	920	7.98	<.0001
Hyperlipidemia	342	0.74	263	2.28	<.0001
Diabetes	758	1.64	252	2.19	<.0001
COPD	187	0.41	91	0.79	<.0001
Asthma	334	0.72	142	1.23	<.0001
Stroke	502	1.09	284	2.46	<.0001
IHD	1083	2.35	476	4.13	<.0001
Pneumonia	817	1.77	860	7.46	<.0001
ESRD	79	0.17	74	0.64	<.0001
Pulmonary Embolism	42	0.09	123	1.07	<.0001
**Mean of follow-up years (SD)** ^#^	6.84 (3.04)		6.47 (3.21)		<.0001

SLE, systemic lupus erythematosus; COPD, chronic obstructive pulmonary disease; IHD, ischemic heart disease; ESRD, end-stage renal disease; chi-squared test;

^#^Student *t* tests.

**Fig 3:**
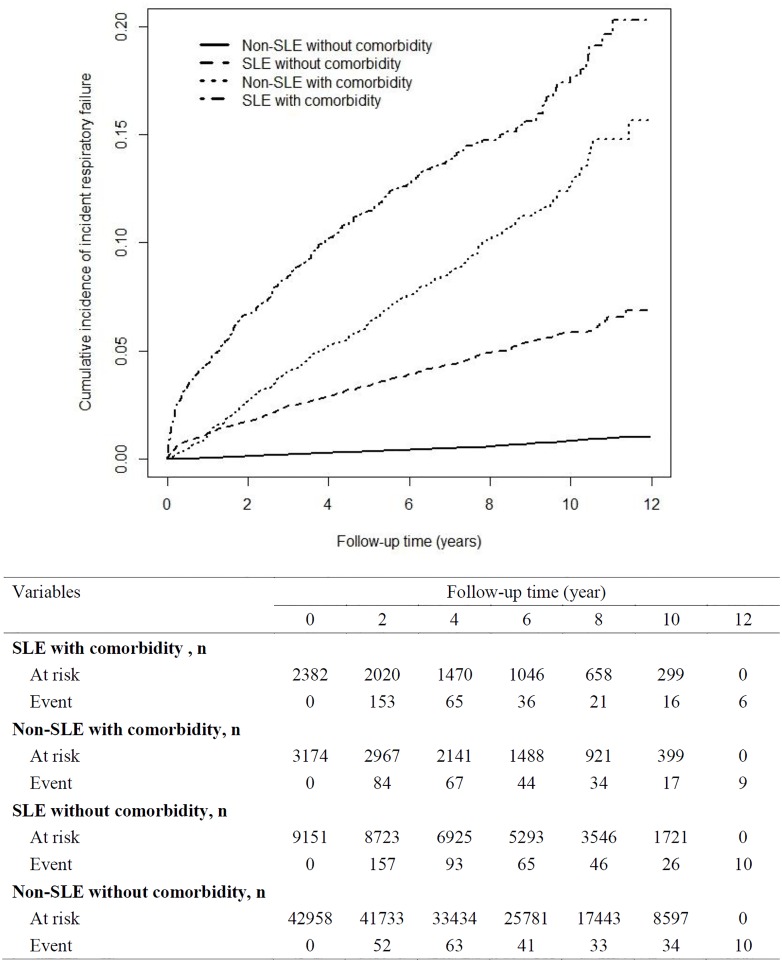
Cumulative incidence of incident respiratory failure in the different subgroups.

In the SLE cohort, the overall IRR for incident respiratory failure was 6.02 (95% CI = 5.72–6.33) and the aHR was 5.80 (95% CI = 5.15–6.52; Table 2). In the sex-stratified analysis, the women with SLE exhibited a 6.79-fold (95% CI = 5.93–7.77) increased risk of incident respiratory failure compared with the women without SLE. Moreover, men with SLE exhibited a 3.44-fold (95% CI = 2.67–4.43) increased risk of incident respiratory failure compared with the men without SLE. The incidence rate of incident respiratory failure increased with age. Regardless of age group (<35, 35–65, or ≥65 y), patients with SLE had an increased risk of incident respiratory failure than did those without SLE (<35 y: aHR = 31.2, 95% CI = 21.6–45.2; 35–65 y: aHR = 6.19, 95% CI = 5.09–7.54; ≥65 y: aHR = 2.35, 95% CI = 1.92–2.87). In both cohorts, the incidence rate of incident respiratory failure was higher among the patients with pre-existing comorbidities. Among those without pre-existing comorbidities, the risk of incident respiratory failure was 9.08-fold (95% CI = 7.72–10.7) higher in the SLE cohort than the non-SLE cohort. Among those with pre-existing comorbidities, the SLE cohort still exhibited an increased risk of incident respiratory failure compared with the non-SLE cohort (aHR = 2.65, 95% CI = 2.22–3.15) after adjustment for potential risk factors. Fig 3 shows the cumulative incidence of incident respiratory failure in the 4 subgroups: 1) the SLE with pre-existing comorbidities, 2) the non-SLE with pre-existing comorbidities, 3) the SLE without pre-existing comorbidities and 4) the non-SLE without pre-existing comorbidities. Fig 3 display the highest cumulative incidence of incident respiratory failure was in the SLE with pre-existing comorbidities.

**Table 2:** Incidence and adjusted HR of incident respiratory failure between the non-SLE and SLE cohorts, stratified by sex, age, and comorbidity.

	SLE							
		No			Yes		Compared to non-SLE cohort	
Variables	Event	PY	Rate	Event	PY	Rate	IRR (95% CI)	Adjusted HR (95% CI)
**Overall**	488	315622	1.55	694	74616	9.30	6.02(5.72–6.33)**	5.80(5.15–6.52)***
**Sex**								
Women	346	279566	1.24	579	66279	8.74	7.06(6.68–7.46)***	6.79(5.93–7.77)***
Men	142	36056	3.94	115	8337	13.8	3.50(3.04–4.04)***	3.44(2.67–4.43)***
**Age, year**								
<35	32	168804	0.19	264	41205	6.41	33.8(30.2–37.8)***	31.2(21.6–45.2)***
35–65	164	129503	1.27	280	30076	9.31	7.35(6.78–7.97)***	6.19(5.09–7.54)***
≥65	292	17314	16.9	150	3335	45.0	2.67(2.26–3.15)***	2.35(1.92–2.87)***
**Comorbidity**								
No	233	296729	0.79	397	61409	6.46	8.23(7.78–8.71)***	9.08(7.72–10.7)***
Yes	255	18893	13.5	297	13206	22.5	1.67(1.45–1.91)***	2.65(2.22–3.15)***

SLE, Systemic Lupus Erythematosus; PY: person-year; Rate: incidence rate (per 1,000 person-y); IRR, incidence rate ratio; HR, hazard ratio; multiple analysis including age, sex, and comorbidities;

**P < 0.01,

***P < 0.001.

Table 3 presents the comorbidity-stratified analysis. Compared to the non-SLE cohort, the incidence of incident respiratory failure was higher in those without pre-existing comorbidities (All *P* values < .001) among the SLE cohort. Meanwhile, the incidence of incident respiratory failure was higher in those with pre-existing comorbidities except ESRD and pulmonary embolism among the SLE cohort also (All *P* values < .001; except ESRD and pulmonary embolism).

**Table 3:** Incidence and adjusted HR of incident respiratory failure between the non-SLE and SLE cohorts, stratified by comorbidity type.

	SLE							
		No			Yes		Compared to non-SLE cohort	
Variables	Event	PY	Rate	Event	PY	Rate	IRR (95% CI)	Adjusted HR (95% CI)
**Hypertension**								
No	351	309067	1.14	548	70062	7.82	6.89(6.53–7.26)***	6.78(5.91–7.78)***
Yes	137	6555	20.9	146	4553	32.1	1.53(1.24–1.90)***	2.90(2.25–3.74)***
**Hyperlipidemia**								
No	463	313686	1.48	660	73119	9.03	6.12(5.81–6.44)***	5.76(5.10–6.50)***
Yes	25	1936	12.9	34	1496	22.7	1.76(1.14–2.72)**	5.53(3.04–10.1)***
**Diabetes**								
No	388	311580	1.25	642	73489	8.74	7.02(6.66–7.40)***	6.55(5.76–7.45)***
Yes	100	4041	24.8	52	1127	46.2	1.87(1.38–2.52)***	2.34(1.64–3.33)***
**COPD**								
No	452	314718	1.44	670	74231	9.03	6.28(5.97–6.62)***	6.06(5.37–6.85)***
Yes	36	904	39.8	24	385	62.4	1.57(0.88–2.78)	2.47(1.39–4.41)***
**Asthma**								
No	456	313781	1.45	661	73909	8.94	6.15(5.85–6.48)***	5.90(5.23–6.67)***
Yes	32	1841	17.4	33	707	46.7	2.69(1.71–4.22)***	3.46(2.06–5.81)***
**Stroke**								
No	408	313045	1.30	642	73301	8.76	6.72(6.38–7.08)***	6.38(5.62–7.25)***
Yes	80	2576	31.1	52	1314	39.6	1.27(0.90–1.81)	2.32(1.58–3.40)***
**IHD**								
No	361	308495	1.17	622	71742	8.67	7.41(7.02–7.82)***	7.08(6.19–8.08)***
Yes	127	7126	17.8	72	2874	25.1	1.41(1.09–1.81)**	2.11(1.57–2.85)***
**Pneumonia**								
No	452	311281	1.45	588	70098	8.39	5.78(5.49–6.08)***	5.85(5.17–6.63)***
Yes	36	4340	8.29	106	4518	23.5	2.83(2.08–3.85)***	4.21(2.83–6.27)***
**ESRD**								
No	478	315240	1.52	683	74193	9.21	6.07(5.77–6.39)***	5.86(5.20–6.61)***
Yes	10	381	26.2	11	423	26.0	0.99(0.46–2.14)	1.96(0.69–5.56)
**Pulmonary Embolism**								
No	480	315360	1.52	678	73949	9.17	6.02(5.72–6.34)***	5.90(5.24–6.65)***
Yes	8	262	30.5	16	667	24.0	0.79(0.37–1.66)	1.26(0.45–3.52)

SLE, systemic lupus erythematosus; COPD, chronic obstructive pulmonary disease; IHD, ischemic heart disease; ESRD, end-stage renal disease; PY: person-year; Rate: incidence rate (per 1,000 person-y); IRR, incidence rate ratio; HR, hazard ratio; multivariable analysis including age, sex, and comorbidities;

**P < .01,

***P < .001.

Table 4 presents the association between the risk of incident respiratory hortfailure and the number of medical visits per year by patients with SLE. The risk of incident respiratory failure was highest in the SLE patients with more than 24 outpatient visits and hospitalizations (aHR = 21.7, 95% CI = 18.0–26.1). The risk of incident respiratory failure was higher in patients who had a higher annual medical visit and hospitalization frequency.

**Table 4:** Adjusted HR of incident respiratory failure associated with the annual frequency of outpatient visits and hospitalizations because of SLE exacerbation.

Variables	N	Event	Rate	Crude HR (95% CI)	Adjusted HR (95% CI)
**Non-SLE cohort**	46132	488	1.55	1.00	1.00
**Number of outpatient visits and hospitalizations per year**					
≤ 11	5302	178	4.85	3.14(2.65–3.73)***	3.09(2.60–3.67)***
12–17	3965	173	6.63	4.28(3.60–5.09)***	4.77(4.00–5.69)***
18–24	1548	173	19.5	12.4(10.4–14.7)***	11.4(9.57–13.7)***
> 24	718	170	58.0	35.8(30.1–42.7)***	21.7(18.0–26.1)***
*p-value for trend*				<.0001	<.0001

SLE, Systemic Lupus Erythematosus; HR, hazard ratio; multivariable analysis including age, sex, and comorbidities;

***P < .001.

The propensity score-matching analysis [35] was used to reduce bias when investigating the association between incident respiratory failure outcomes and SLE. In total, 9841 and 29 523 patients were included in the SLE and non-SLE cohorts, respectively (Table 5). The standardized difference was low in the baseline potential risk in both cohorts (standardized difference < 0.1) [35]. After propensity score matching, the risk of incident respiratory failure remained higher in the SLE cohort than in the non-SLE cohort (aHR = 7.84, 95% CI = 5.82–10.6; Table 6).

**Table 5:** Comparison in demographic status and health status between SLE and non-SLE cohorts after propensity score matching.

	SLE				
	No (N = 29523)		Yes (N = 9841)		Standardized difference
Variables	n	%	n	%	
**Sex**					0.002
Women	26257	88.9	8759	89.0	
Men	3266	11.1	1082	11.0	
**Age, year**					0.002
<35	16500	55.9	5501	55.9	
35–65	11435	38.7	3897	39.6	
≥65	1588	5.38	443	4.50	
Mean (SD) ^#^	34.8 (15.7)		34.6 (15.2)		0.002
**Comorbidity**					
Hypertension	368	1.25	95	0.97	0.027
Hyperlipidemia	163	0.55	55	0.56	0.001
Diabetes	573	1.94	188	1.91	0.002
COPD	102	0.35	46	0.47	0.019
Asthma	188	0.64	71	0.72	0.010
Stroke	208	0.70	126	1.28	0.058
IHD	546	1.85	273	2.77	0.062
Pneumonia	134	0.45	49	0.50	0.006
ESRD	41	0.14	20	0.20	0.016
Pulmonary Embolism	2	0.01	2	0.02	0.012

SLE, systemic lupus erythematosus; COPD, chronic obstructive pulmonary disease; IHD, ischemic heart disease; ESRD, end-stage renal disease.

**Table 6:** Incidence and adjusted hazard ratio of incident respiratory failure between non-SLE and SLE cohorts by propensity score matching.

	SLE							
		No			Yes		Compared to non-SLE cohort	
Variables	Event	PY	Rate	Event	PY	Rate	IRR (95% CI)	Hazard Ratio (95% CI)
**Overall**	271	204242	1.33	488	65457	7.46	5.62(5.27–5.99)***	7.84(5.82–10.6)***

SLE, systemic lupus erythematosus; PY: person-year; Rate: incidence rate (per 1,000 person-years); IRR, incidence rate ratio;

***p<0.001.

## Discussion

This study revealed that regardless of age, sex, or pre-existing comorbidities, the incidence of incident respiratory failure was higher in the SLE cohort than in the non-SLE cohort (aHR = 5.80, 95% CI = 5.15–6.52). SLE patients older than 65 years of age exhibited a higher incidence of incident respiratory failure, which is in contrast to the poor prognosis of young women [37] reported in a previous study [18]. A possible explanation may be that older patients in the SLE and non-SLE cohorts tended to have more primary complications, such as cardiovascular [11] and pulmonary lesions [38], resulting from the primary deterioration [38,39] of the patients in the SLE cohort; these primary complications may be risk factors for the onset of incident respiratory failure among older adults.

In this study, males [aHR = 3.44, Table 2] with SLE exhibited a higher risk of incident respiratory failure as did their female [aHR = 6.79,Table 2] counterparts [40]. Furthermore, the incidence of incident respiratory failure was higher in patients with SLE without pre-existing comorbidities than it was in those without SLE and pre-existing comorbidities [Fig 3]. The risk of incident respiratory failure in the SLE cohort without pre-existing the atherosclerosis-related diseases (e.g.; hypertension [aHR = 6.78], stroke [aHR = 6.38], IHD [aHR = 7.08]; Table 3) were still higher. These findings aid clinicians by revealing that men with SLE but without pre-existing comorbidities [25,41] could develop incident respiratory failure.

Systemic lupus erythematosus is protean in its manifestations and follows a relapsing and remitting course. No study has investigated the relationship of the numbers of SLE exacerbations and related hospital admissions with the risk of incident respiratory failure. We observed that an increased frequency of outpatient department visits and hospitalizations was associated with SLE exacerbation. This finding suggests that poor control of the deterioration of an obstructed airway, occlusion of the pulmonary vessel, and atherosclerosis of the arterial system or coronary artery were crucial factors for incident respiratory failure in the SLE cohort. The recurrence of SLE exacerbation causes primary cumulative damage [39] to the lung parenchyma (e.g., pneumonia) [19,42], airway (e.g., airway obstruction [12,26,43,44]), and pulmonary vessel (e.g., pulmonary embolism) [13,45], thus resulting in hypoxemia and incident respiratory failure [29] in SLE patients without pre-existing comorbidities.

Several factors may predispose patients with SLE to respiratory failure, including pulmonary lesions, such as those in the airway [12], vessels [19], interstitium [11], and pleura [41]; and extrapulmonary lesions such as those in the brain [11], kidneys [10], and heart [11]. In patients with SLE, the functions of these organs progressively deteriorate [46], which is associated with incident comorbidities [18,45,47]. In our study, the patients with SLE without pre-existing comorbidities (e.g., pulmonary embolism [aHR = 5.90], ESRD, [aHR = 5.86]; Table 3) had a higher risk of incident comorbidities with respiratory failure compared with the non-SLE cohort; this finding concurs with that of a previous study [48].

If patients with SLE develop pulmonary or cardiovascular lesions along with the pre-existing pulmonary disease (e.g., airway, parenchyma [12]), microvascular disease (e.g., diabetes [16,18]), or macrovascular disease (e.g., hypertension, stroke) [12,15,17], the dual effects may contribute to cumulative damage to the airway or system vessels [39,49], thereby increasing the risk of incident respiratory failure [3,29]. In our study, the SLE patients with pre-existing comorbidities (e.g., hypertension, [aHR = 2.90], diabetes [aHR = 2.34], COPD [aHR = 2.47], stroke [aHR = 2.32], and pneumonia [aHR = 4.21]; Table 3) exhibited a higher risk of the incident respiratory failure compared with the non-SLE cohort; this finding is in line with the findings of previous research.

Our findings alert clinicians to be aware of the effect of SLE on the risk of incident respiratory failure [29,42], particularly in patients who frequently receive medical services, regardless of age, sex, and pre-existing comorbidities.

## Limitations

This study has several limitations that should be addressed when interpreting its findings. The NHIRD does not provide detailed lifestyle information, such as smoking history, body mass index, or physical activity level, all of which are potential confounding factors for this study. However, the SLE treatment and lifestyle modification of patients with SLE may implicate these factors in accelerated respiratory lesions in SLE. In addition, information on the severity of SLE, such as disease activity, functional impairment, and physical damage, may result in the underdiagnosis of hypertension, hyperlipidemia, or diabetes. Patients listed in the RCIPD receive resgular immunosuppressant therapy. The lack of detailed drug data, such as hydroxychloroquine and glucocorticosteroids, for adjusting the outcomes of interest could be another limitation. In addition, the clinical presentation of SLE may vary. Undiagnosed SLE [50] may affect the diagnosis of incident respiratory failure. Pleural lesions and interstitial lung disease are potential factors for respiratory failure [3]. In addition, SLE patients complicated with antiphospholipid Ab syndrome may be at a higher risk of developing respiratory failure [51]. Hypercoagulation state due to formation of antiphospholipid Ab can result in catastrophic diseases resulting from vascular occlusion in such diseases as stroke (n = 284), IHD (n = 476), ESRD (n = 74), and pulmonary embolism (n = 123) (Table 1). We could identify only approximately 8.3% of possible cases of aniphospholippid Ab syndrome from the current SLE cohort (n = 11 533 in Table 1). There is no specific ICD-9 code for antihopholipid Ab syndrome that could have assisted us in exploring this issue in the current study. Moreover, hemorrhagic alveolitis [52] is a rare disease [47] complicated with the antihopholipid Ab syndrome, which was not analyzed in the present study. The diagnosis of pneumonia [53] requires thoracic imaging, titer of the serum, sputum smear/culture, respiratory specimens, or tissues with pathological proof [42], which were not available in this study [54]. Finally, the prevalence of COPD [55] in young adults [56] is low [57], and, from an epidemiological perspective, the incidence of asthma is relatively lower in control groups. Asthma and COPD may indicate past respiratory failure [58] before the SLE diagnosis date. Therefore, patients with asthma or COPD were excluded from our study if they had past respiratory failure before the index date (SLE diagnosis date), which may explain the relatively lower frequency of asthma in the non-SLE group; thus, this is another confounding factor. These limitations warrant further investigation.

## Strengths

In Taiwan, SLE is categorized as a catastrophic disease [12]; thus, the validity period of this disease is valid for life. Each patient in this study was individually followed up until the diagnosis of incident respiratory failure. In addition, the professional discipline care of SLE patients since 2007 is in line with the 1995–2007 period. Therefore, the coverage of the insurance program after 2007 is similar to that from 1995 to 2007, with no significant differences [59]. Furthermore, respiratory failure is also categorized as a catastrophic disease in Taiwan [60]. These 2 diseases should be scrutinized through peer review by a specialist [12]. Multidisciplinary teams (e.g., rheumatologist, cardiologist, pulmonologist, respiratory therapist and certified educator) guide the assessment, treatment, and holistic care of patients with incident SLE and subsequent incident respiratory failure [12,61]. This study adopted a nationwide population-based cohort longitudinal design to evaluate the risk of incident respiratory failure in a mostly Asian population with SLE. The findings can thus be generalized to the general population [62].

## Conclusion

This study determined that patients with SLE are associated with an increased risk of incident respiratory failure, regardless of age, sex, and pre-existing comorbidities; this increased risk is particularly pronounced among patients who frequently receive medical services.

## Supporting Information

S1 Checklist (DOC)

## Abbreviations

aHR: adjusted hazard ratio
CI: confidence interval
NHIRD: National Health Insurance Research Database
NHI: National Health Insurance
NHRI: National Health Research Institutes
LHID 2000: Longitudinal Health Insurance Database 2000
SLE: systemic lupus erythematosus
COPD: chronic obstructive pulmonary disease
ESRD: end-stage renal disease
ICD-9-CM: International Classification of Diseases, Ninth Revision, Clinical Modification
